# The Effectiveness of Group-Based Core Stability Exercise and Educational Booklet for Hospital Workers in Taiwan with Nonspecific Low Back Pain: A Preliminary Study

**DOI:** 10.3390/ijerph19063324

**Published:** 2022-03-11

**Authors:** Ching-Yueh Lin, Yung-Hsuan Liu, Shu-Mei Chen, Su-Chun Cheng, Mei-Fang Liu

**Affiliations:** 1 Department of Physical Medicine and Rehabilitation, Kaohsiung Armed Forces General Hospital, Kaohsiung 802301, Taiwan; linchingyueh@gmail.com (C.-Y.L.); me12347@gmail.com (Y.-H.L.); 2 Physical Medicine and Rehabilitation Division, School of Medicine, National Defense Medical Center, Taipei 114201, Taiwan; 3 Department of Physical Therapy, College of Health Science, Kaohsiung Medical University, Kaohsiung 807378, Taiwan; shumei@gap.kmu.edu.tw; 4 Department of Medical Research, Kaohsiung Medical University Hospital, Kaohsiung Medical University, Kaohsiung 807378, Taiwan; 5 Department of Physical Therapy, School of Medical and Health Science, Fooyin University, Kaohsiung 831301, Taiwan; ptsuchun@gmail.com

**Keywords:** disability, early management, exercise therapy, low back pain, occupational health, pain, quality of life, workplace

## Abstract

To investigate the effectiveness of health promotion strategies for nonspecific low back pain in hospital workers, we compared the therapeutic effects of group-based core stability exercises and an educational booklet. Subjects participated in a 60-min core stability exercise on a weekly basis for 8 weeks (*N* = 24) or consulted an educational booklet for advice (*N* = 22). The numerical rating scale (NRS), Oswestry Disability Index (ODI), and the brief version of the World Health Organization’s Quality of Life (WHOQOL-BREF) were used as outcome measures. The ODI, as well as the total score and domains of overall, physical, and psychological health in the WHOQOL-BREF were significantly improved in the exercise group (*p* < 0.05). The NRS score significantly improved in the booklet group (*p* < 0.05). The total score, psychological domain, and environmental domain of the WHOQOL-BREF improved significantly in the exercise group compared with the booklet group (*p* < 0.05). Group-based core stability exercises and educational booklets are helpful to hospital workers in different ways for nonspecific low back pain. In contrast to the pain reduction by the educational booklet, more active participation in group-based core stability exercise can provide a better outcome in the overall quality of life, especially in the psychological and environmental domains of hospital workers.

## 1. Introduction

A previous study reported that more than 80% of adults have encountered low back pain in their lifetime [1]. Most low back pains are self-limited or recover quickly; however, approximately 33% of them recur within one year and could progress to chronic low back pain [2]. According to the National Institute for Occupational Safety and Health in the United States, back injury is one of the most common occupational injuries [3]. According to the US Bureau of Labor Statistics (2001), the incidence rates of musculoskeletal symptoms among nursing professionals are roughly twice that of the national mean in a 15-year time frame [4]. Medical personnel in Taiwan also suffered from low back pain at 60.9–85.1% prevalence rate, depending on occupational categories [5,6,7]. A study from Taiwan indicated that higher severity of low back pain might affect the working performance of nurses [8]. Approximately 87.4% of nurses reported that their work efficiency and daily life were not affected by low back pain in a Taiwan study, and only 20% of them needed doctor visits or medication treatments. Approximately 65.9% of nurses considered that low back pain was sourced from work [6].

Many methods address occupational low back pain, such as physical therapy, exercise, multidisciplinary rehabilitation, counseling, and education booklets [9]. Rantonen et al. (2018) indicated that multidisciplinary rehabilitation (comprehensive care including medical care, physical medicine and rehabilitation, physical or occupational therapy, behavioral counseling, and vocational component dealing) and exercises were equivalent in eliminating low back pain, but multidisciplinary rehabilitation was infrequently used because of its labor-intensive and time-intensive nature [10]. In a systematic review and meta-analysis, it was verified that core stability exercise provided more improvement in pain and disability for low back pain than general exercise (strengthening, stretching, and aerobic exercises) [11]. According to a meta-analysis study, there was no significant difference between the therapeutic effects of group-based or individual physiotherapy exercise programs on musculoskeletal pain and low back pain [12]. The educational booklet is commonly used among interventions for low back pain, alone, with verbal review [10,13], or in combination with physical exercise training [14]. It also proved effective in managing occupational low back pain by offering a back information booklet, regardless of an additional booklet review [10,13].

To the best of our knowledge, this study is the first to address the outcome differences between core stability exercises and patient education booklets [13,15,16,17]. This study aimed to compare the therapeutic effects of core stability exercises and the patient education booklet, uncover limitations in practicality, and provide further information in clinical practice.

## 2. Materials and Methods

### 2.1. Study Design and Sample

This study used convenience sampling, a between-subjects design, and a non-randomized control group pretest-posttest design. Patients were enrolled from the staff of the Kaohsiung Armed Forces General Hospital. The inclusion criteria were staff who were aged ≥20 years and had low back pain screened by the musculoskeletal symptoms questionnaire [18], which was modified from the Nordic Musculoskeletal Questionnaire [19]. The exclusion criteria included the presence of either analgesic use or physical therapy, diagnoses of low back pain (e.g., lumbar spondylosis, herniated disc disease, spondylolisthesis), any previous lumbar spine surgery, severe musculoskeletal disorders (e.g., deformities in the lower limbs and lumbar spine), systemic connective diseases, mental disorders, and pregnancy. This study was approved by the Institutional Review Board of Kaohsiung Armed Forces General Hospital (KAFGHIRB 109-004). Informed patient consent was obtained.

### 2.2. Study Protocol

**Figure 1:**
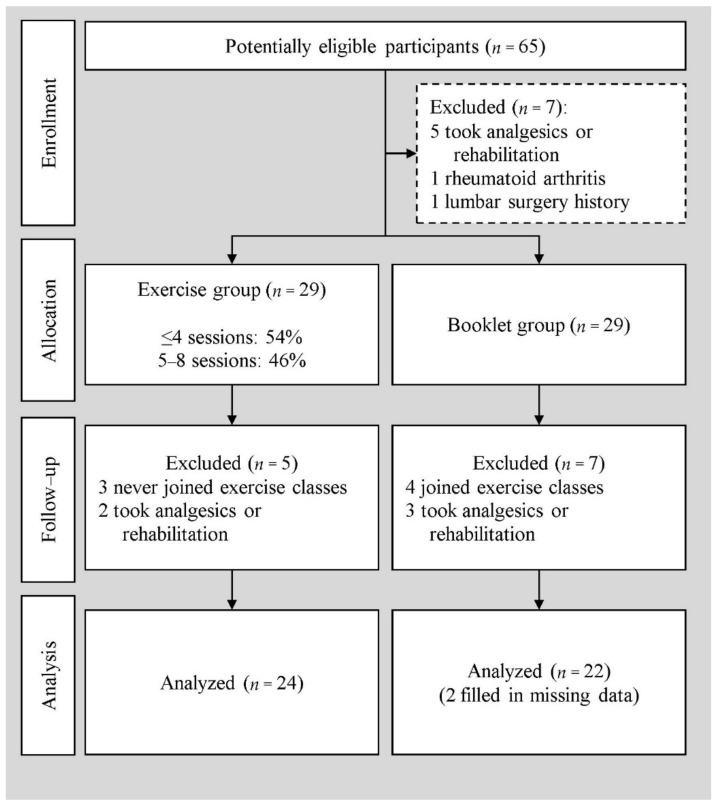
Flowchart.

Upon enrollment, the participants were informed of the study protocol, and their demographic data were registered. Pretests were simultaneously performed on all participants. Participants could self-decide whether to join the exercise group or the booklet group. Pretests were performed 1 month before the exercise intervention, while posttests were completed 1 month after the exercise intervention. Figure 1 shows the flowchart.

In this study, 65 of the hospital staff members were included after excluding five on analgesics, one diagnosed with rheumatoid arthritis, and one who had undergone lumbar surgery in the past. Twenty-nine cases were in the exercise and booklet groups, respectively. A total of eight core stability exercise sessions were implemented between 10 March 2021 and 12 May 2021. The schedule was rearranged twice because of weather issues. Before the posttest, five patients were excluded from the exercise group due to non-participation in the exercise course (3/5) and the use of analgesics or physical therapy (2/5); seven patients were excluded from the booklet group due to participation in the exercise program (4/7) and the use of analgesics or physical therapy (3/7). Finally, 24 cases in the exercise group and 22 in the booklet group were included in the analysis. The posttest data for two individuals in the booklet group were missing, and the missing data replacement method was used for these two datasets (Figure 1).

### 2.3. Intervention

Exercise group: The schedule and location of the core stability exercise program were set to be convenient for all participants. The program was held at a basketball stadium in the hospital after working hours. Each session was 60 min in duration at weekly intervals. A coach with 17 years of teaching experience adopted suggestions from Akuhota, Hicks, and Rabin [20,21,22] to design the program. The group exercise and interspersed Pilates and jumping exercises in the program were aimed at increasing fun and participation. Exercise content and intensity were tailored using feedback from the participants. The contents of the core stability exercise program are presented in Table S1.

Booklet group: Information on the Occupational Low Back Pain Prevention Booklet issued by the Taiwan Institute of Labor, Occupational Safety and Health, Ministry of Labor, includes an introduction to low back pain, preventative methods for low back pain in daily activities, and treatments for low back pain. The back book was provided and explained to the participants by the investigators.

### 2.4. Outcome Measures

#### 2.4.1. Numerical Rating Scale

The numerical rating scale (NRS) is used to quantify a subjective characteristic, which is difficult to measure directly, ranging across a continuum of scores. A score of 0 indicates no pain and a score of 10 indicates an extreme amount of pain. Respondents point to positions on the scale that correspond to their agreement on the level of low back pain in 1 month. The NRS has good reliability with an intraclass correlation coefficient (ICC) of 0.83 [23]. The minimum clinically important difference for chronic musculoskeletal pain is a reduction of one point or a reduction of 15% in the scale [24].

#### 2.4.2. Oswestry Disability Index-Chinese Version 2.1

The Oswestry Disability Index—Chinese version 2.1 (ODI) [25] is a self-checked questionnaire with ten topics in terms of pain intensity, personal care, lifting, walking, sitting, standing, sleeping, social life, sexual activity, and traveling. For each topic, six statements were presented as different scenarios relating to the topic. The patient checks the closest statement that has happened and resembles real life. Each question is scored on a scale of 0–5: 0 indicates the least amount of disability and 5 indicates maximal disability. The index score is then derived from the sum of the scores for each topic question, which is then multiplied by 2 (total of 0–100). A sum of 0 indicates no disability, and 100 indicates extreme disability. The topic of sexual activity can be omitted, giving a final maximum sum of 90. The index score can also be translated as a percentage (index score/total score × 100%). A range of 0–20% means the least disability, and a range of 80–100% means extreme disability. The convergent validity of the Chinese ODI was supported by its high correlation with the Roland Morris Disability Questionnaire (r = 0.76). The ICC of the test-retest reliability was 0.89 [26]. For low back pain, the optimal cutoff point is 9.5 [24] and the minimum detectable change is 6% [27].

#### 2.4.3. WHOQOL-BREF Taiwan Version

Of the 28 questions in the brief version of the World Health Organization’s Quality of Life (WHOQOL-BREF) Taiwan version, two evaluated general health, and seven, six, four, and nine questions evaluated physical health, psychological, social relationships, and environment domains, respectively. The subtotal score in each domain was divided by the number of questions and then multiplied by 4, resulting in scores of 4–20. The sum of the subtotal scores for each domain then falls between 28 and 140. The higher the score, the better the quality of life is [28]. The instrument has been previously verified to have good reliability and validity [29].

### 2.5. Statistical Analysis

IBM SPSS 20.0 was used for all data analyses. The Shapiro–Wilk test was used to test the outcome variables, and Levene’s test was used to test the homogeneity of the variables. The paired samples *t*-test was used when the intragroup pretest-posttest differences were normally distributed; otherwise, the Wilcoxon signed-rank test was used. The independent samples *t*-test was used for intergroup comparisons when the outcome variables were normally distributed; otherwise, the Mann–Whitney U test was used. The chi-squared test was used for categorical variables. A two-tailed test was used, and a significant difference was set at *p* < 0.05. Missing values were replaced by using a linear trend with a medium.

## 3. Results

Basic demographic data are shown in Table 1. There were no significant differences between the two groups in terms of their scores in the musculoskeletal symptoms questionnaire, duration, sex, age, job tenure, body height, body weight, body mass index, education, job type, dominant hand, smoking habits, and exercise habits. The mean participation was 4.25 sessions in the core stability exercise program, and the training adherence was 53%.

**Figure 2:**
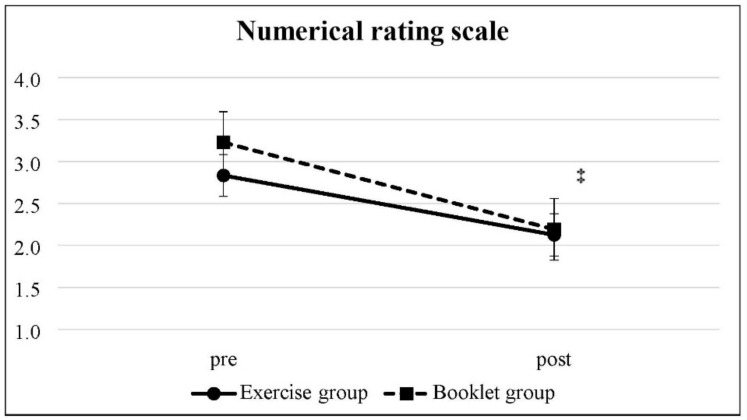
Numerical rating scale: Mean scores with 95% confidence intervals at the pretest and posttest. ^‡^ Significant difference between pretest and posttest in the booklet group.

**Figure 3:**
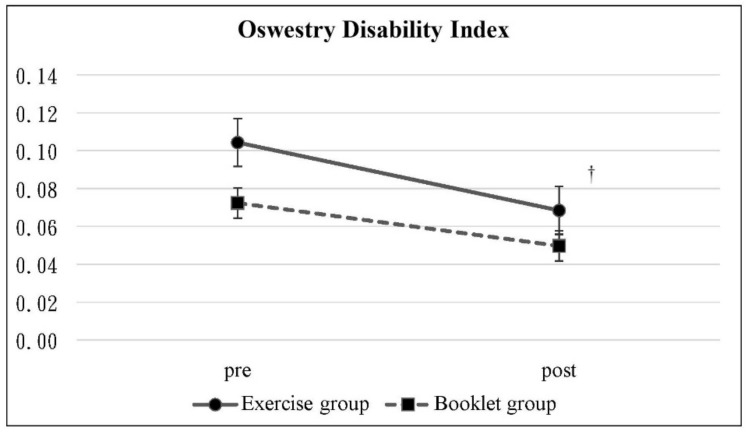
Oswestry Disability Index: Mean scores with 95% confidence intervals at the pretest and posttest. ^†^ Significant difference between pretest and posttest in the exercise group.

**Figure 4:**
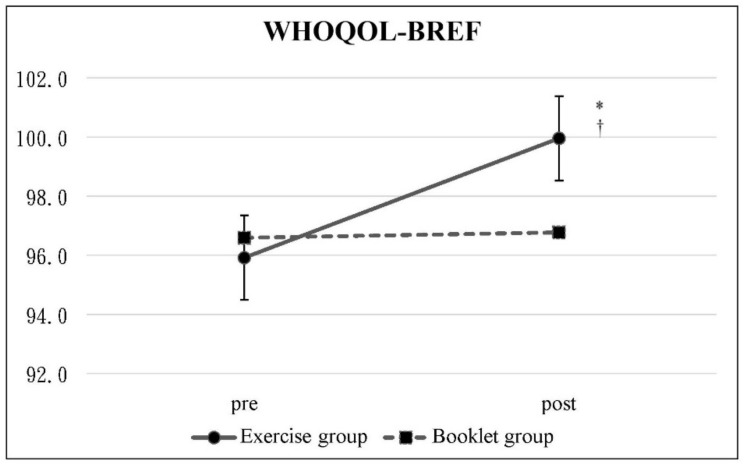
WHOQOL-BREF: Mean scores with 95% confidence intervals at the pretest and posttest. ^†^ Significant difference between pretest and posttest in the exercise group. * Significant difference between the two groups.

The data from pretest and posttest are shown in Table 2. The change in NRS score was statistically significant in the booklet group (*p* = 0.01) but not in the exercise group (*p* > 0.05) (Figure 2). In the exercise group, the ODI percentage dropped significantly from 10.43 to 6.84% (*p* = 0.04) (Figure 3), and the WHOQOL-BREF scores increased significantly from 95.92 to 99.92 (*p* = 0.01) (Figure 4), in which the scores in the overall, physical health, and psychological domains were also statistically significant. In contrast, there was no significant change in the ODI and WHOQOL-BREF scores in the booklet group. The intergroup comparison showed significant differences in the WHOQOL-BREF and psychological and environmental domains of the exercise group compared to that of the booklet group (*p* < 0.05) (Figure 4).

## 4. Discussion

According to our results, we found that the 8-weekly-session core stability exercise was beneficial for the disability level and quality of life of hospital staff with nonspecific low back pain. Meanwhile, we noted that an education booklet for low back pain was also helpful in pain reduction. However, the improvement in quality of life was more significant in the exercise group than in the booklet group, although there are no obvious differences in pain reduction and disability index in the exercise group.

The design of our intervention method was based on the designs of other studies, with some modifications. In the core stability exercise intervention, we added elements such as Pilates and jumping to the exercise course. The therapeutic effect of Pilates on chronic non-specific low back pain was proven in a review [30]. Additionally, heavy resistance training to the back extensor muscles, traditional sit-ups, and full flexion or repetitive torsion of the lumbar spine may be injurious to the lumbar spine [20]; therefore, such activities were absent in the exercise design to avoid harmful effects on the lower back.

There are several reasons for the insignificant pain reduction in the exercise group. First, our participants were selected using a screening tool for the musculoskeletal symptoms; therefore, the severity of the pain may be lower than that of real patients in the clinic. Participants undergoing analgesic treatment or physical therapy were also excluded. The NRS score of 2.83 in our study was much lower than the 5.18 in the study by Noormohammadpour et al. [16]. The disability index of 10.43 in our study was also less than 18 in the study by Rasmussen-Barr et al. [31] and 24.89 in the study by Sung [32]. In this case, the level of pain in our participants in the initial setting was relatively low, which may result in insignificant pain reduction. Second, training adherence directly affects therapeutic efficacy. Our results had a training adherence rate of 53%. Jakobsen et al. (2015) demonstrated a training adherence of 45% and a response rate for pain reduction of 80% by joining a work-based exercise course compared to a training adherence of 21% and a response rate of 42% by joining a home-based exercise course [33]. Third, the disease stages were mixed among our participants: 17%, 8%, 71%, and 4% in the acute, subacute, chronic, and unknown stages, respectively. Hayden et al. stated that exercise was beneficial in pain reduction and functionality for chronic low back pain; however, it was not significantly advantageous for acute and subacute low back pain compared to no treatment or other treatments [34]. The mixed disease stages of low back pain in our study may have resulted in underestimation of the therapeutic effect of core stability exercises. Despite this, the author suggested applying exercise-based therapy in all cases of subacute and chronic low back pain [35]. Lastly, the weekly exercise course was rescheduled twice, and we could not estimate how much it interfered with the therapeutic effect.

There are four models in designing education booklets for low back pain, including the biomedical model, biopsychosocial model, individualized biomechanical model, and pain neurophysiology education [36,37,38]. However, therapeutic effects for low back pain by using education booklets were inconsistent in pain and functionality [13,36,39]. This study is the first to employ a prevention booklet for occupational low back pain, which is issued by Taiwan’s Institute of Labor, Occupational Safety, and Health, Ministry of Labor and uses the biopsychosocial model. Our results showed significant pain improvement in the booklet group but not in the disability index and quality of life. Henrotin et al. (2006) suggested that a biopsychosocial booklet could provide information about low back pain and shift a patient’s beliefs about body activities, pain, and consequences of low back pain [40]. Another author also indicated that educational materials for non-labor-intensive workers could facilitate behavioral modifications, health beliefs and attitudes, and psychological distress secondary to health issues at work [41]. An educational booklet not only provides information about lower back pain but also enhances patients’ beliefs in practical modifications to the work environment and activities, which can explain the successful pain reduction by a booklet in our study.

This is the first study to compare the therapeutic effects between core stability exercise therapy and an education booklet. In our results, exercise therapy had a better outcome in terms of quality of life than an education booklet but had no better outcomes for pain reduction and disability index. The studies by Zou et al. (2021) and Ferreira et al. (2021) were designed to evaluate the add-on effect of exercise therapy combined with health consultation. Zou et al. showed that core stability exercise and health education together provided more effects on pain reduction and functional improvement than health education alone [17]. Ferreira et al. also found that exercise therapy and an educational booklet were equivalent in terms of outcome benefits [15]. Quality of life was not one of the outcome measures in their studies [15,17], but it was greatly improved by core stability exercise therapy in our study. Participants with low back pain benefited more from group-based exercise than home-based exercise in terms of personal well-being, work satisfaction, desire to exercise, and energy in engagement with family and friends [33]. This may explain why better quality of life was achieved in the exercise group.

In our study, we noted the significant therapeutic effect of core stability exercise training over only eight weeks to help reduce low back pain among the hospital staff. The limitations of this study include the design of a non-randomized controlled trial and subjective outcome measurements that may have a bias from the Hawthorne effect. Second, pain level being low (NRS) at the starting point may lead to an insignificant therapeutic effect of the exercise intervention. In addition, the power for quality of life was only 0.56. Further large-scale studies are needed in the future.

## 5. Conclusions

For non-specific occupational low back pain among hospital staff, core stability exercise therapy provides the greatest benefit in improving the quality of life. Nonetheless, offering an educational booklet remains an effective and convenient solution to reduce low back pain.

## Figures and Tables

**Table 1:** Demographic data at the baseline.

	Exercise Group (*n* = 24)	Booklet Group (*n* = 22)	*p*-Value
Musculoskeletal symptoms questionnaire (1/2/3/4/5) ^a,e^	10/7/3/4/0	4/13/3/1/1	0.131
Pain duration (1 month/3 month/over 6 month/unannotated) ^a,e^	4/2/17/1	6/1/11/4	0.291
Sex (male/female) ^a,f^	2/22	1/21	0.607
Age (year) ^b,c^	45.50 ± 10.74	41.95 ± 11.85	0.293
Job tenure (year) ^b,c^	19.24 ± 12.25	16.79 ± 11.39	0.481
Height (cm) ^b,c^	160.38 ± 7.27	159.05 ± 5.64	0.495
Weight (kg) ^b,d^	60.40 ± 7.22	60.02 ± 14.16	0.904
Body mass index (kg/m^2^) ^b,c^	23.55 ± 2.92	24.42 ± 4.68	0.448
Education (high school/junior college/university/master/doctor) ^a,e^	1/1/18/4/0	4/5/12/1/0	0.060
Job type (long sitting/long standing/half) ^a,e^	6/3/15	8/0/14	0.198
Dominant hand (left/right) ^a,f^	0/24	1/21	0.296
Smoking (no/yes) ^a,f^	23/1	22/1	0.950
Exercise habit (No exercise/occasionally/1–2 times a week/3–5 times a week) ^a,e^	3/9/10/2	2/14/3/3	0.159
Mean participation ^b^	4.25 ± 2.56	-	-

^a^ Number of individuals; ^b^ mean ± SD; ^c^ independent samples *t*-test; ^d^ Mann–Whitney U test; ^e^ chi-square test; ^f^ Fisher’s exact test.

**Table 2:** Outcome scores at the pretest and posttest.

	Exercise Group (*n* = 24)			Booklet Group (*n* = 22)			P2 ^c^	Effect Size
	Baseline	After	P1 ^b^	Baseline	After	P1 ^b^		
Numerical rating scale ^a^	2.83 ± 2.14	2.12 ± 1.99	0.09	3.23 ± 1.41	2.19 ± 1.18	0.01 *	0.54	0.18
Oswestry Disability Index (%) ^a^	10.43 ± 10.79	6.84 ± 7.72	0.04 *	7.23 ± 10.44	4.79 ± 5.82	0.17	0.57	−0.27
WHOQOL-BREF								
Total score ^a^	95.92 ± 13.10	99.96 ± 12.61	0.01 *	96.59 ± 10.44	96.77 ± 11.15	0.88	0.04 *	0.64
Overall ^b^	12.33 ± 2.41	13.50 ± 2.15	0.03 *	13.27 ± 2.00	13.97 ± 2.05	0.14	0.43	0.22
Physical health ^a^	14.14 ± 2.16	15.10 ± 1.68	0.01 *	14.36 ± 1.62	14.76 ± 1.83	0.13	0.18	0.40
Psychological ^a^	13.19 ± 2.41	14.03 ± 2.17	0.005 *	13.21 ± 1.72	13.04 ± 1.78	0.55	0.01 *	0.77
Social relationships ^a^	14.29 ± 1.73	14.17 ± 1.79	0.17	14.00 ± 1.72	13.69 ± 1.45	0.78	0.75	0.14
Environment ^a^	13.74 ± 2.13	14.04 ± 2.22	0.09	13.78 ± 2.03	13.64 ± 1.97	0.29	0.04 *	0.37

P1 = *p*-value compared within a group; P2 = *p*-value compared between the two groups; ^a^ mean ± SD; ^b^ paired samples *t*-test or Wilcoxon rank signed test; ^c^ independent samples *t*-test or Mann–Whitney U test; * *p* < 0.05.

## Data Availability

The data presented in this study are available on request from the corresponding author. The data are not publicly available for the protection of patients’ privacy.

## Supplementary Materials

The following supporting information can be downloaded at: https://www.mdpi.com/article/10.3390/ijerph19063324/s1, Table S1. The content of core-stability exercise program.
