# Supplementary material for: The Effectiveness of Group-Based Core Stability Exercise and Educational Booklet for Hospital Workers in Taiwan with Nonspecific Low Back Pain: A Preliminary Study
Source: Int J Environ Res Public Health. 2022 Mar 11;19(6):3324. doi: 10.3390/ijerph19063324 (PMC8954112; doi:10.3390/ijerph19063324)
Supplement: Supplementary file 1 [file ijerph-19-03324-s001.zip › ijerph-1549041-supplementary.pdf]

International Journal of
Environmental Research and Public Health

MDPI

Supplementary Material

Table S1. The content of core-stability exercise program.

Core-Stability Exercise Program								
	**Day 1**	**Day 2**	**Day 3**	**Day 4**	**Day 5**	**Day 6**	**Day 7**	**Day 8**
Basis principle of Pilates: Breathing, pelvic placement, rib cage placement, scapular movement and stabilization, head and cervical placement.	V (supine)	V (supine)		V (sitting)	V (sitting)			
Supine position								
Glute bridge	V	V	V	V	V	V	V	
Pelvic tilt	V	V	V					
Partial curl			V	V	V			
Toe touch				V	V	V	V	
Supine bicycle					V			
Single leg bridge						V	V	
Dead bug						V	V	
Prone position								
Swimmers	V	V	V					
Forearm plank				V	V	V	V	V
Plank body saw				V	V			
Plank single leg saw				V	V			
Plank crawl					V	V	V	V
High plank						V	V	V
Plank up down							V	V
Panther shoulder tap								V
All four position								
Bear holds			V			V	V	V
Mountain climber						V	V	V
Bull dog							V	V
Bear plank walk								V
Standing position								
Squat	V	V	V	V	V			
Side lunge	V	V	V			V	V	

Alternating side lunge		V	V			V	V	
Slide shuffle		V	V			V	V	
Carioca		V	V					
Calf raises	V	V	V					
High kicks			V					
Jump squats			V	V	V			
Standing core rotation			V	V	V			
Oblique twist						V	V	
Foot fire						V	V	V
Burpee jacks							V	V
Foot fire—lateral							V	V
Jumping jacks							V	V
Lateral jumping jacks								V

Sixty minutes per session (including 10 min of warm-up and 5 min of cool-down); 2–3 sets × 5–10 reps for each as tolerated.
